# Using technology to engage hospitalised patients in their care: a realist review

**DOI:** 10.1186/s12913-017-2314-0

**Published:** 2017-06-06

**Authors:** Shelley Roberts, Wendy Chaboyer, Ruben Gonzalez, Andrea Marshall

**Affiliations:** 1 NHMRC Centre of Research Excellence in Nursing, Menzies Health Institute Queensland, Griffith University, Gold Coast Campus, Gold Coast, QLD 4222 Australia; 2 School of Information and Communication Technology, Griffith University, Gold Coast Campus, Gold Coast, QLD 4222 Australia; 3 School of Nursing and Midwifery, Menzies Health Institute Queensland, Griffith University, Gold Coast Campus, Gold Coast, QLD 4222 Australia; 4 Nursing and Midwifery Education and Research Unit, Gold Coast University Hospital, Southport, QLD 4215 Australia

**Keywords:** Beside technology, Complex interventions, Health care interventions, Health information technology, Hospital care, Patient participation, Patient-centred care, Patient engagement, Realist review, Technology

## Abstract

**Background:**

Patient participation in health care is associated with improved outcomes for patients and hospitals. New technologies are creating vast potential for patients to participate in care at the bedside. Several studies have explored patient use, satisfaction and perceptions of health information technology (HIT) interventions in hospital. Understanding what works for whom, under what conditions, is important when considering interventions successfully engaging patients in care. This realist review aimed to determine key features of interventions using bedside technology to engage hospital patients in their care and analyse these in terms of context, mechanisms and outcomes.

**Methods:**

A realist review was chosen to explain how and why complex HIT interventions work or fail within certain contexts. The review was guided by Pawson’s realist review methodology, involving: clarifying review scope; searching for evidence; data extraction and evidence appraisal; synthesising evidence and drawing conclusions. Author experience and an initial literature scope provided insight and review questions and theories (propositions) around why interventions worked were developed and iteratively refined. A purposive search was conducted to find evidence to support, refute or identify further propositions, which formed an explanatory model. Each study was ‘mined’ for evidence to further develop the propositions and model.

**Results:**

Interactive learning was the overarching theme of studies using technology to engage patients in their care. Several propositions underpinned this, which were labelled: information sharing; self-assessment and feedback; tailored education; user-centred design; and support in use of HIT. As studies were mostly feasibility or usability studies, they reported patient-centred outcomes including patient acceptability, satisfaction and actual use of HIT interventions. For each proposition, outcomes were proposed to come about by mechanisms including improved communication, shared decision-making, empowerment and self-efficacy; which acted as facilitators to patient participation in care. Overall, there was a stronger representation of health than IT disciplines in studies reviewed, with a lack of IT input in terms of theoretical underpinning, methodological design and reporting of outcomes.

**Conclusion:**

HIT interventions have great potential for engaging hospitalised patients in their care. However, stronger interdisciplinary collaboration between health and IT researchers is needed for effective design and evaluation of HIT interventions.

## Background

Patient engagement, also known as patient participation in health care, is a worldwide patient safety priority. Patient participation in care is endorsed by the World Health Organisation [1] and national health care accreditation bodies in the USA [2], Australia [3], UK [4] and many other countries. Patient participation in care is associated with improved patient safety, fewer adverse events, improved healthcare behaviours and outcomes, and higher patient satisfaction with care [5, 6]. Patients may participate in their care to different extents, and in different ways; including information sharing, self-management and shared decision making.

Advances in health information technology (HIT) are creating opportunities for patients to actively engage in care in a variety of ways, which is expected to improve quality and cost-effectiveness of health care [7]. A recent systematic review of 170 studies found technology-based health interventions had positive effects on patient engagement, health behaviours and health outcomes among patients across a range of conditions [7]. Reviews have synthesised evidence around the effectiveness of HIT interventions such as patient portals [8, 9], decision support aids [10] or multiple technologies [7, 11, 12] for patient engagement in health care in the primary care setting. Together these reviews suggest great potential for HITs to engage patients in care, but overall highlight a lack of high quality evidence on their effectiveness in terms of health outcomes and cost-effectiveness.

Fewer studies have explored the use of HIT for patient participation in care in the hospital setting [13], despite strong directives for patient engagement [3]. Only one review of 17 studies has evaluated HIT interventions in hospitals, highlighting research on the use of technology for inpatient participation in care is still in its infancy [13]. Nonetheless, emerging evidence shows promise for HIT interventions to benefit patients, staff and organisations; such as by improving access to health information, enhancing communication, streamlining processes and enabling patient participation in care [14–16].

Many factors may determine whether interventions using technology to engage patients in hospital care are successful, and these should be considered from both health and information technology (IT) perspectives. When designing interventions, health care providers (HCPs) consider the aims and content; yet effectiveness of human computer interfaces encompasses not only the information or functionality provided to the end user, but how it is provided (i.e. form vs. function). A review of 41 HIT interventions for health behaviour change highlighted the importance of interdisciplinary collaborations between computer, health and behaviour sciences in the development, implementation and evaluation of HIT interventions [12]. A better understanding of the theories of adult learning, patient engagement, behaviour change, and educational/interactive IT are likely to result in better informed interventions that can be meaningfully evaluated. The review also found most studies did not identify which aspects of HIT interventions contributed to outcomes [12]. The UK’s Medical Research Council recommends complex interventions undergo process evaluation to understand the relationship between context, mechanisms and outcomes (CMO); that is, what works for whom under what conditions [17]. The only review of interventions using technology to engage patients in care in the inpatient setting described key themes of HIT interventions, but did not explore the CMO relationship to understand what aspects of interventions worked, why, how and for whom [13]. Previous reviews also lack an interdisciplinary approach, as they have only considered HIT interventions from a health provider perspective (focusing on theories grounded in health) and not from an IT perspective (i.e. theories of educational technology and IT usability); limiting their usefulness to researchers and clinicians looking to design and evaluate such interventions.

Realist reviews explore the CMO underpinning interventions [18], making them a valuable tool in designing, evaluating and interpreting complex interventions. This realist review aimed to determine key features of interventions using bedside technology to engage hospital patients in their health care and analyse these in terms of context, mechanisms and outcomes, using an interdisciplinary approach.

## Methods

A realist review was selected to explain how and why complex HIT interventions work or fail within certain contexts, settings or populations; hence, it has an explanatory rather than a judgemental approach [18]. There is a focus on the relationship between context, mechanism and outcome; that is, how context affects the mechanism of action underlying an intervention to generate a certain outcome. As health service delivery in the hospital setting is complex, multifaceted, dynamic and heterogeneous, the same intervention may work differently in different settings or contexts [19]. Pawson’s realist review methodology was used to guide this study and involved several steps, which are outlined below. This approach involved theorising about why and how interventions worked in different contexts.

### Clarify scope

This realist review started with consideration of the review question, which unlike systematic review, was an ongoing and iterative process [18]. We drew on experience and conducted an initial scope of the literature to identify key terms, concepts and theories that provided some insight into the use of HIT to engage hospitalised patients in their care. Steps taken and considerations for clarifying the scope of the review are outlined in Table 1.

**Table 1:** Clarify scope (Step 1) in the realist review process guided by Pawson et al. (2005)

Key step	Considerations/sub-steps	Actions for current review
Identify review question	Nature and content of the intervention	Interventions using technology, available at the bedside, to engage patients in their hospital care for management or prevention of a health concern were the focus of this review. The initial review questions were ‘What are the features of successful interventions using technology to engage hospitalised patients in their health care?; Why are these features important?; and In what contexts do they work?’
	Circumstances or context for its use	
Refine purpose of review	Theory integrity: does the intervention work as predicted?	Authors’ theories for why interventions worked were identified and evaluated in an initial exploratory search of the literature. The theorised mechanisms of action in studies were evaluated to identify those that seemed to result in patient engagement in care. Intervention strategies and underlying mechanisms of action were compared among different settings and patient populations to identify what worked for whom under what conditions.
	Theory adjudication: which theories fit best?	
	Comparison: how does the intervention work in different settings, for different groups?	
Articulate key theories to be explored	Draw up a ‘long list’ of relevant programme theories by exploratory searching	An initial exploratory search of the literature, for the explicit purpose of identifying ‘the theories, the hunches, the expectations, the rationales and the rationalisations for why the intervention might work’ was conducted. Theories behind why interventions worked were collected from a number of papers and put into a ‘long list’ of key intervention theories. These were grouped into categories based on similarity; this formed a basic, provisional model (which was later refined) to help focus the literature search and guide initial data extraction.
	Group, categories and synthesise theories	
	Design a theoretically based evaluative framework to be ‘populated’ with evidence	

### Search for evidence

**Fig. 1:**
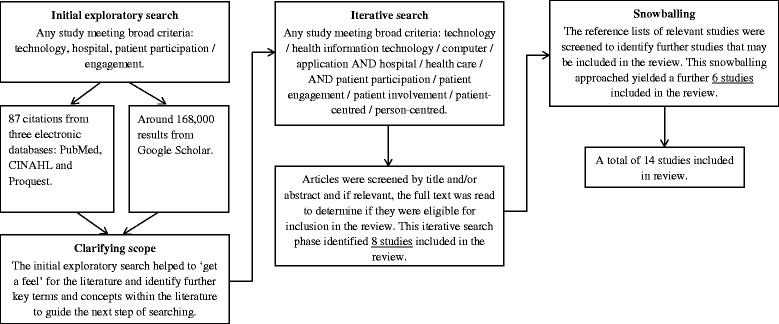
Flow diagram illustrates the search strategy, based on the RAMSES realist syntheses publication standards [44]

After initial exploratory searches were conducted to ‘get a feel of the literature’ and identify theories (or propositions) behind HIT interventions (as outlined in Table 1), a more focused and purposive literature search was conducted to find evidence to support or refute propositions [18]. A final search was conducted once synthesis was almost complete to seek out additional studies that might further refine the explanatory model [18]. Databases searched included PubMed, Cumulative Index of Nursing and Allied Health Literature (CINAHL), ProQuest and Google Scholar. Search terms included various combinations of the following key words: application, computer, engagement, health care, health information technology, hospital, involvement, participation, patient(s), patient-centred, person-centred, technology. Articles published after 2004 were included, as technology prior to this time was considered to be outdated. Studies were included if they were conducted in the hospital setting, involved patient participants, used some form of bedside technology to engage patients in their health care and reported patient-focussed outcomes. Due to the limited literature available in the area, patient population was not restricted. Both quantitative and qualitative studies were included. Studies were excluded if they were not published as full-text articles, did not report any outcomes (i.e. only reported the design of a technology), or were commentaries or discussion papers. Snowball sampling was used to identify further papers, from reference lists or ‘cited by’ lists of already included studies. Searching ceased when there was sufficient evidence to answer the research question (i.e. when data saturation was reached) Fig. 1.

### Data extraction and evidence appraisal

Unlike systematic reviews, the quality of evidence in realist synthesis is not judged on the methodological design hierarchy (i.e. with RCTs as gold standard), as multiple methods are required to explain what, when, how and why interventions work [18]. In this review, studies were considered by their relevance (whether they addressed the propositions under test) and rigour (whether they were methodologically credible, in terms of outcomes reported and methods used, for testing propositions). A tool was developed to extract data such as context, setting, participants, intervention, postulated theories as to why it did or didn’t work, and outcomes. Each study was read in detail, and relevant data were highlighted, noted and entered into a table, guided by the data extraction tool. Papers were ‘mined’ for evidence that contributed to further development of the propositions in the explanatory model [18, 20]. That is, papers were searched for ideas or theories on how the intervention was supposed to work. These were highlighted, noted and given an approximate label. The different labels or theories were roughly grouped together and formed propositions, and each paper reviewed further contributed to the development of the model and propositions. Data extraction was iterative in that studies were returned to at different points throughout the review process [18].

### Synthesising evidence and drawing conclusions

The purpose of the review drove the synthesis process. The included studies were considered in relation to the explanatory model to see which propositions remained relevant and the model and propositions were iteratively revised and refined to best explain the data. Each proposition was considered in terms of CMO (i.e. the proposed mechanism of action to achieve an outcome and how context may have affected these), using evidence from a range of studies. Reflection and theorising resulted in multiple propositions, and studies could have encompassed none, one or more of them.

## Results

Fourteen studies were included in this review, outlined in Table 2. From the studies reviewed, an overarching theme and five propositions emerged, explaining the features of and mechanisms behind interventions using technology to engage patients in their hospital care. Patient-centred outcomes including patient satisfaction with and use of HIT, and patients’ perceived usefulness and usability of HIT were reported; as the majority of studies were feasibility or usability studies (i.e. none reported clinical outcomes). However as the purpose of this review was to identify features of HIT interventions that successfully engage patients in their hospital care, these patient-centred outcomes were appropriate to assess as they are likely to influence overall participation in care using HIT.

**Table 2:** Context, mechanisms and outcomes for each proposition

Proposition 1: Information sharing allows patients to be better informed about their health condition and health care, facilitating participation in care through improved communication, patient empowerment, informed decision making, self-care and self-management.					
Author, year, country	Context		Mechanisms		Outcomes
	Setting	Participants	Intervention (tools/resources)	Proposed action	
(Wilcox, Woollen et al. 2016) [30], USA^a^	30-bed post-operative cardiac unit, university hospital, New York.	20 cardio-thoracic surgery patients, mean age 58 (range 26–81) years, 40% female; and *n* = 2 family members.	Personal health record portal accessed via iPad provided to patients. Included: patient-specific information (demographics, care providers, medication orders and records) and educational content (cardiovascular conditions, tests and procedures).	Providing access to health information improves patients’ knowledge and awareness; communication with HCPs; self-care; and participation. Patient safety may be improved as patients can resolve discrepancies in data.	70% were active users (used program unsupervised). 90% found it useful. Patients liked ‘patient notepad’ for communicating with HCPs and liked ability to share information with family.
(O’Leary, Lohman et al. 2015) [27], USA^b^	General medical units, large academic hospital in Chicago.	100 medical patients, mean age 47 years, 63% female, 44% Caucasian.	Patient portal accessed via iPad provided to patients. Included: general patient information, care team, medication list and agenda for the day (i.e. mealtimes and scheduled tests and procedures).	Providing access to health information improves patients’ knowledge in some areas. Unfamiliar terminology, inadequate time to review portal, lack of motivation or preferring a passive role in care may be barriers to technology.	High portal use (80% used). High satisfaction (76% found it easy to use, 71% reported information was useful). Patients had greater knowledge of their doctors’ names and roles but no significant improvements in other areas.
(Cook, Moradkhani et al. 2014) [26], USA^c^	Mayo clinic hospital	149 elective cardiac surgery patients aged >50 (mean 68) years, expected LOS 5–7 days.	Patient recovery program, accessed via iPad provided by hospital. Included: hospital plan of stay, education, recovery planning, daily ‘to do’ lists, and daily self-assessment and reporting (discharge planning and mobility screening tools).	Information sharing helps patients understand their condition and plan of care, which improves participation in care and self- management.	High completion (84% of 6295 modules delivered were completed; mean 42 modules per patient). High understanding (90% of patients understood 90% of content before discharge).
(Greysen, Khanna et al. 2014) [24], USA^d^	Hospital (details not reported)	30 medical patients (60% aged ≥40 years).	Web-based interactive health education modules and personal health records; accessed via iPads provided to patients. Educational modules included: medication safety, handwashing, falls prevention, communicating with HCPs, discharge planning. Personal health record contained functions to view/refill medications, view /modify appointments, send messages to HCPs.	Providing access to health information promotes patient engagement in discharge planning through increased knowledge about plan of care.	High satisfaction (90% satisfied or very satisfied). High engagement/use of program (83% independently completed modules).
(Dykes, Carroll et al. 2013) [28], USA^e^	General medical units in two academic medical centres	8 patients and 3 family members on general medical units, aged 37–90 (mean age 64) years.	Electronic bedside communication centre accessed via internet on secure mobile tablet device provided to patients. Contained: orientation to environment (hospital, care team); schedule (events, consultations, tests, mealtimes); notes (patients/family could document questions/concerns to discuss with HCPs); and health information (medication schedule, test results, tailored education).	Providing patients with information about their condition and opportunity to discuss this with HCPs is a strategy for engaging patients in their recovery plan and shared decision making.	High satisfaction with program and information contained, particularly around their care (i.e. care team, medications, test results, health condition information etc.). Wanted ability to communicate with HCPs from the bedside and order meals online.
(Pfeifer Vardoulakis, Karlson et al. 2012) [15], USA^f^	Emergency department in an urban hospital	25 patients and 8 family (mean age 46 years, 92% African-American).	Private access to own information through a mobile phone (provided to patients). Contained: key events (i.e. room assignment, medication administration); profile (general and demographic information); care team (names, titles, pictures of HCPs); and medications and tests, with brief explanations of each item and ability for patients to email pages to themselves or their GP.	Information sharing improves patients’ and families’ knowledge and awareness of their condition and care, which enables participation in care through empowerment, shared decision making and self-management.	High usage (22 of 25 used regularly). High satisfaction; patients felt less anxious as they were more aware of their condition and care plan. They found medical explanations useful and liked sharing information with their families. Access to the phone helped them remember details they would otherwise forget.
(Vawdrey, Wilcox et al. 2011) [16], USA	Step-down cardiology unit at a large urban academic medical centre	5 male patients (mean age 55, mean LOS 5 days) and one spouse.	Personal health record portal accessed through iPad (provided to patients). Contained personal health information (demographics, HCPs, medication orders); information to help patients understand their conditions, tests and procedures; and information targeted at cardiovascular health.	Sharing of health information via iPads enables participation in care through increased knowledge, adherence and shared decision making.	Patients were satisfied and felt engaged in care process. They perceived the program to be useful; liked ability to review information at own pace; felt more connected with team; felt less burdened to remember details of admission; liked to review accuracy of information in electronic record.
Proposition 2: Self-assessment and feedback enhances patient learning through interactivity, embedding of knowledge and relevance and specificity of information provided. This learning results in increased patient empowerment and responsibility in their care and improved communication between patients and HCPs, which facilitates patient participation in care.					
(Tzeng, Yin et al. 2015) [31], USA^g^	Adult subacute stroke rehab inpatient unit.	5 patients aged ≥65 years (falls risk)	Web-based software application accessed through an android tablet device (provided to patients). Contained: inpatient fall risk assessment; set of approaches and tasks selected by patients, that they can do themselves to prevent falls; ability to create individualised falls prevention plan for printing.	Self-assessment of falls risk helps patients understand their own risk factors. Self-selection of tasks patients can do themselves to minimise each risk factor helps them to make decisions to reduce falls risk. This empowers patients to be informed, active and collaborative partners in their care.	Patients liked the design of Patients liked the design of iEngaging as (1) an easy to use and effective tool; (2) allowing patient to tailor falls prevention and self-management care plan; (3) giving patient a voice and facilitating increased communication with health care providers.
(Cook, Manning et al. 2013) [32], USA^h^	Mayo clinic hospital	149 elective cardiac surgery patients aged >50 (mean 68) years, expected LOS 5–7 days.	Patient recovery program, accessed via iPad provided by hospital. Included: hospital plan of stay, education, recovery planning, daily ‘to do’ lists, and daily self-assessment and reporting (discharge planning and mobility screening tools).	Patient self-assessment and self-reporting, integrated into an individualised plan for hospital recovery, increases patients’ responsibility for a shared plan of care; facilitates patient input into care; and enables patients to actively participate in their care.	High completion rates: 1,384 of 1,418 self-assessment modules delivered to 149 patients were completed (97.6%).
(Oosterom-Calo, Abma et al. 2014) [23], UK ^i^	Cardiology ward at a UK academic hospital.	7 male heart failure patients aged 37–76.	Motivate4Change: interactive health promotion program, accessed via a tablet computer (provided to patient). Contained introduction, medication adherence and physical activity modules. Each module contained: introduction, list of key messages, video with information on each topic, showing a ‘typical’ heart failure patient dealing with issues (i.e. role modelling), and assessments and feedback.	Self-assessments of knowledge, barriers and beliefs around medication adherence and physical activity allow for tailored feedback messages that are targeted and relevant to patients, and hence more likely to be acceptable and perceived as useful.	Patients enjoyed interactive learning and felt it better addressed their learning needs. Patients appreciated receiving targeted information and felt their personal situations were acknowledged through the assessments and feedback, which seemed to encourage them to perform healthy behaviours.
(Ruland, Holte et al. 2010) [29], Norway	Norwegian university hospital.	145 patients starting leukaemia/ lymphoma treatment, mean age 49–50 years.	Interactive module on iPad (provided to patients) where patients could communicate symptoms, concerns, problems to HCPs and prioritise these. Tailored questions and recommendations given based on patients’ initial responses.	Patient-completed assessments of symptoms, problems and concerns, which are prioritised and then shared with HCPs, enables patient empowerment and participation in care through improved communication with HCPs and shared care planning.	Intervention group: significantly more symptoms and problems addressed by HCPs; greater reductions in symptoms distress; greater reduction in need for symptom management support compared to control.
Proposition 3: Patients are more accepting of, engaged in and satisfied with education that is tailored to reflect their personal situation and information needs, as information is perceived to be useful and relevant. Knowledge gained through engaging in tailored education empowers patients to take greater responsibility for and participate in their care.					
(Cook, Moradkhani et al. 2014) [26]	^c^As above		^c^As above	Tailored education helps patients understand their condition and plan of care, and identify and manage potential complications; and is a means to increased self-management, adherence and satisfaction. Education and recovery planning modules, tailored to patients’ surgery and medical conditions, empowers patients to share ownership of and participate in care.	5,267 of 6295 education modules (84%) delivered to 149 patients were completed (mean 42 ± 3 modules completed per patient). Completion rates were higher in men than women, but were not associated with age. 90% of patients indicated they understood 90% of the content prior to discharge.
(Oosterom-Calo, Abma et al. 2014) [23]	^i^As above		^i^As above	Education tailored to patients’ existing knowledge, barriers and beliefs; needs and preferences; medical condition and personal situation is more likely to be relevant, useful and acceptable to patients. Patients are more likely to adhere to the self-management strategies learned if they perceive them to be relevant, useful and acceptable.	Patients had high levels of satisfaction; thought the program was contextual, empathic and relevant to their life and situation. They felt encouraged, motivated and comforted by the program. They appreciated receiving less information that was more specific and relevant to them; however all patients read all of the information provided, with some expressing a positive attitude towards learning.
(Bickmore, Mitchell et al. 2010) [21], USA ^j^	Urban academic safety net hospital	131 patients (19 with major depressive condition), mean age 46–49.	Computer-animated conversational agent (virtual nurse) accessed via a wheeled kiosk with a touchscreen. Provides information prior to hospital discharge on: post-discharge care regimen (medications, follow up appointments, exercise and diet, test results), supplemented with a booklet.	Education about patients’ diagnosis and post discharge self-care regimen, tailored to patients’ medical conditions, health literacy and empathic needs, is more likely to be understood, acceptable and useful to patients with special needs (i.e. with low health literacy or depressive symptoms). When patients understand information and find it to be acceptable and useful, they are more likely to use it.	Very high reported satisfaction, ease of use and attitudes towards agent. Only 24% of patients would have preferred information from doctor or nurse. Appreciated amount of time the agent spent with them. Patients with inadequate health literacy and major depressive symptoms reported a greater therapeutic alliance with the agent than patients with adequate health literacy and no depressive symptoms.
(Bickmore, Pfeifer et al. 2009) [21], USA^k^	3 inpatient floors of a medical centre.	19 patients, 53% female, aged 25–75 (mean age 55), 42% with inadequate health literacy.	Computer-animated conversational agent (virtual nurse) accessed via a wheeled kiosk with a touchscreen. Provides information prior to hospital discharge on: post-discharge care regimen (medications, follow up appointments, exercise and diet, test results), supplemented with a booklet.	Education about patients’ diagnosis and post discharge self-care regimen, tailored to patients’ medical condition and health literacy, enables patient participation in care by empowering them (through increased knowledge) to self-manage their own health. This increased knowledge also empowers patients to communicate with HCPs and engage in shared decision making.	All participants completed interaction with no problems. Patients were highly satisfied and found it easy to use, informative, and useful/helpful. Patients felt comfortable receiving health information from a computer and reported they would follow advice given by virtual nurse. Patients appreciated amount of information and time given by virtual nurse (i.e. more than HCPs provide). 60% of patients chose to hear additional details/cover more content. Patients felt empowered by the information they received.
Proposition 4: A user-centred design, that is, incorporating user perspectives (such as patients and clinicians) in the design of HIT aiming to engage patients in their hospital care is essential for developing programs that meet patients’ information and learning needs that are also acceptable to and used by patients. If end-users are involved in design and development of HIT, they are more likely to be engaged in using the program, as it is relevant and acceptable to them.					
Author, year, country	Context	Mechanisms^*^		Outcomes	
(Wilcox, Woollen et al. 2016) [30]	^a^As above	A user-centred process of aligning program design with information needs of cardio-thoracic surgery patients and ensuring interaction and presentation techniques of the tools match abilities and preferences of target population maximise patient satisfaction, acceptability and use of the program.		Patients had high levels of satisfaction (90% were satisfied) and use (70% were active users) and found the program useful and easy to use.	
(O’Leary, Lohman et al. 2015) [27]	^b^As above	Patients’ preferred content for the portal was identified in a prior study; but no mechanism was described for how this related to outcomes.		^b^As above; but not linked with mechanisms of user-centred design.	
(Oosterom-Calo, Abma et al. 2014) [23]	^i^As above	A user-centred design approach incorporating user perspectives, needs and preferences is essential for patients to actually use the system, and for it to be successful.		Patients had high levels of satisfaction and thought the program was contextual, empathic and relevant to them. They felt the information provided met their needs.	
(Dykes, Carroll et al. 2013) [28]	^e^As above	An iterative participatory software development process was used, incorporating end-user needs and perspectives, which was proposed to enhance patients’ understanding and use of the technology.		Patients were highly satisfied the program and information it contained, particularly around their care team, medications, test results and health information.	
(Pfeifer Vardoulakis, Karlson et al. 2012) [15]	^f^As above	Authors assessed basic usability and intuitiveness of HIT design and conducted a pilot study to gain user feedback, which was incorporated into end design; however no mechanism was described for how this may lead to outcomes.		^f^As above; but not linked with mechanisms of user-centred design.	
Proposition 5: Supporting patients in the use of HIT, including familiarisation, training and ongoing support is critical to patients’ acceptance, engagement and use of this technology. Patients acknowledge that both HCPs and HIT have unique but complementary roles, and both are important for enabling participation in care.					
Author, year, country	Context	Mechanisms^*^		Outcomes	
(Wilcox, Woollen et al. 2016) [30]	^a^As above	15 min training and observations session was provided; however no mechanism was described for how this may lead to outcomes.		^a^As above; but not linked with mechanisms of support in HIT use.	
(O’Leary, Lohman et al. 2015) [30]	^b^As above	Patients were given a brief orientation to the program and given contact information for one-on-one support; however no mechanism was described for how this may lead to outcomes.		^b^As above; but not linked with mechanisms of support in HIT use.	
(Tzeng, Yin et al. 2015) [31]	^g^As above	Patients were given 5 min orientation; however no mechanism was described for how this may lead to outcomes.		Patients liked the design of the program but desired time to review the content with a HCP or family member, and desired additional instructions or directions about using the program.	
(Cook, Manning et al. 2013) [32]	^h^As above	Patients were instructed on program use by nurses (no further details given); however no mechanism was described for how this may lead to outcomes.		^h^As above; but not linked with mechanisms of support in HIT use.	
(Greysen, Khanna et al. 2014) [24]	^d^As above	Patients received a tiered orientation tailored to individual experience and needs; however no mechanism was described for how this may lead to outcomes.		^d^As above; but not linked with mechanisms of support in HIT use.	
(Oosterom-Calo, Abma et al. 2014) [23]	^i^As above	Support from HCPs, including explaining the purpose of the program and how to use it, could reduce patient insecurity in using the program and ensure patients are ready and able to participate.		Patients found the program easy to use; even those who expressed negative attitudes towards HIT, however still sought support from others in its use.	
(Pfeifer Vardoulakis, Karlson et al. 2012) [15]	^f^As above	Patients were given a brief (2–4 min) tutorial about the program; however no mechanism was described for how this may lead to outcomes.		^f^As above; but not linked with mechanisms of support in HIT use.	
(Bickmore, Mitchell et al. 2010) [21]	^j^As above	A brief training session was provided to patients; however no mechanism was described for how this may lead to outcomes.		^j^As above; but not linked with mechanisms of support in HIT use.	
(Bickmore, Pfeifer et al. 2009) [21]	^k^As above	A brief training session was provided to patients; however no mechanism was described for how this may lead to outcomes.		^k^As above; but not linked with mechanisms of support in HIT use.	

*GP* General practitioner, *HCP* Health care professional, *HIT* Health information technology, *LOS* Length of stay

^*^Intervention has been described earlier, so ‘Mechanisms’ refers only to mechanisms of action proposed by authors

### Interactive learning facilitates participation in care

**Fig. 2:**
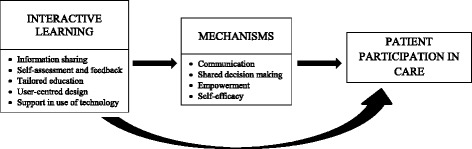
Explanatory model: facilitation of patient participation in care through interactive learning

Interactive learning was the main overarching theme of studies using technology to engage hospitalised patients in their care. There were several propositions underpinning this, which were derived from health or IT ideologies. Figure 2 shows the explanatory model by which interactive learning facilitated patient participation in care and the core concepts related to each proposition. Table 2 describes the CMO for each proposition.

Interactive, self-directed learning through the use of HIT was a feature of all studies, with patients able to control the pace and extent of learning by self-navigating through programs. This provided a low-pressure environment, allowing patients to take as much time as needed to thoroughly understand content [21, 22]. Patients preferred interactive learning than being ‘told’ what to do; they felt it better addressed their learning needs [23]. Interactive learning was thought to enhance patient participation or engagement [21, 23–25] and multiple learning methods were theorised to accommodate for varying learning preferences [26]. Each of the propositions underpinning interactive learning for facilitating patient participation in care are described below in terms of CMO.

**Proposition 1** *Information sharing allows patients to be better informed about their health condition and health care, facilitating participation in care through improved communication, patient empowerment, informed decision making, self-care and self-management.*

Information sharing was a key feature of a number of studies [15, 16, 21, 22, 24, 26–30]. This was done by providing patients with access to their own health information via an electronic health record portal, accessible on a portable device such as a tablet computer, mobile phone or wheeled computer kiosk. Some programs also included functions for secure messaging between patients and HCPs or allowing patients to enter or correct their own health data. Studies using information sharing reported increased patient and family empowerment and engagement in care; increased patient awareness, satisfaction and safety; and improved communication between patients, families and HCPs. These were often intertwined as outcomes and mechanisms; that is, some contributed to others in a theoretical causal chain.

Patient satisfaction was generally very high among studies using information sharing programs [15, 16, 21, 22, 24, 27, 28, 30], as patients found programs informative and useful [15, 21, 22, 27, 30]. They enjoyed being informed about their plan of care [15, 30] and felt more engaged in care processes [16]. Patients expressed that information sharing increased their awareness of ‘what was happening’ and this reduced their anxiety, fear and uncertainty; which in turn resulted in positive patient experiences; particularly in the context of the emergency department [15]. Patients were also highly satisfied with the ability of programs to share information with family [15, 27, 30]; they found it comforting to know their families were informed about their care and able to act as health advocates during hospitalisation [15]. Whilst most studies found patients wanted access to all their health information [15, 16, 24, 28], in some instances patients were overwhelmed by the amount and type of information (such as reading about their medication side effects) [30], indicating information should be tailored to individual preferences. In many studies, patients felt empowered by access to their personal health information, which allowed them to participate more actively in their care, make informed decisions and communicate with HCPs [15, 16, 21]. Information sharing using HIT was also theorised to improve patient safety, as patients used programs to identify and correct discrepancies or errors in data [15, 30]. They also felt greater control over their care [16]. This suggests that providing patients with access to their own health information empowers them to participate in their care and in turn, improve safety and satisfaction with care. Information sharing through HIT is a strategy that may be used to engage patients in their hospital care that is acceptable to patients.

**Proposition 2** *Self-assessment and feedback enhances patient learning through interactivity, embedding of knowledge and relevance and specificity of information provided. This learning results in increased patient empowerment and responsibility in their care and improved communication between patients and HCPs, which facilitates patient participation in care.*

A number of studies used patient self-assessment and immediate feedback as a strategy for interactive learning [21, 23, 26, 29, 31]. This either involved (a) patients completing assessments or questionnaires in order for programs to refine and provide tailored information or education based on patients’ initial responses; or (b) assessment of knowledge gained (and feedback on correctness of answers) after delivery of an education module. Studies using self-assessment and feedback reported high patient engagement in and use of programs; high satisfaction; improved communication between patients and HCPs; patient empowerment and patient participation in care. Several authors theorised self-assessment and feedback enabled programs to deliver information to patients that was relevant and specific, as it was tailored based on patients’ responses. It was also thought to decrease response burden as patients only answered questions that were relevant to them and in return received more targeted information. This was suggested to better meet patients’ learning needs and better engage patients in learning activities. Studies using a baseline self-assessment to identify areas for focusing care delivery theorised that this approach helped patients to understand and communicate their problems and make decisions around their plan of care. It was also thought to improve communication with HCPs and allow patient contribution to care. Other studies using self-assessment and feedback to check patient understanding of information received in programs reported this would help patients in their learning.

Self-assessment and feedback using HIT seems to be a useful tool for (a) assessing patients’ problems or risk factors to assist HCPs in patient-centred care planning; and (b) checking knowledge gained by patients after receiving health information and prioritising human (staff) resources. It also enables patient participation in care through improvements in knowledge and communication; and patient contribution to care planning.

**Proposition 3** *Patients are more accepting of, engaged in and satisfied with education that is tailored to reflect their personal situation and information needs, as information is perceived to be useful and relevant. Knowledge gained through engaging in tailored education empowers patients to take greater responsibility for and participate in their care.*

Most of the HIT interventions reviewed provided individualised information or education to patients, tailored based on their existing knowledge or information needs [21–23, 30], health literacy [21, 22, 30], or health conditions [16, 21–23, 26, 28–30, 32]. Tailored education seemed to result in greater patient satisfaction, acceptability and use of HIT [21, 23, 28]. This may be because patients perceived the information as relevant, useful and valuable. Patients found information useful and easy to understand [21–23, 30] and appreciated receiving less information that was more specific to their needs [23]. The importance of contextual and empathic tailored educational content was emphasised [21–23]. Patients were particularly satisfied when they felt acknowledged, cared about, listened to, and important; such as when more time was spent on them [21, 22] and when information was individualised, personal and contextual to their own life or situation [21–23]. Patients felt empowered by information they received [21, 23], which was theorised to promote participation in care. Tailored education modules had very high completion rates [21, 22, 26, 32] and many studies found patients opted to read or hear additional information (i.e. took the ‘long way’ through programs) [15, 21–23], indicating patients are engaged with technology that provides tailored education.

Tailored education is a means of providing patients with specific, relevant information that is well received and empowers patients to make decisions about and actively participate in care.

**Proposition 4** *A user-centred design, that is, incorporating user perspectives (such as patients and clinicians) in the design of HIT aiming to engage patients in their hospital care is essential for developing programs that meet patients’ information and learning needs that are also acceptable to and used by patients. If end-users are involved in design and development of HIT, they are more likely to be engaged in using the program, as it is relevant and acceptable to them.*

Whilst most studies incorporated aspects of user-centredness, several specifically mentioned a user-centred approach in the design of the program itself [15, 21–23, 28, 30, 33]. In these studies, patients generally had high satisfaction with programs [15, 21–23, 27, 28, 30], perceived the information and content delivered to be relevant and useful [15, 21–23, 27, 30], demonstrated high use [15, 21, 22, 27, 30] and found them easy to use [21, 22, 30]. However, not all studies described mechanisms by which a user-centred design was proposed to contribute to these outcomes. As all studies also used interactive learning strategies (i.e. previous three propositions), it is difficult to determine the extent to which each factor contributed to outcomes such as satisfaction and use. Overall, details of how a user-centred design approach was used, and the CMO relationship for this strategy were difficult to identify in studies.

There are several steps in user-centred design of HIT, known as the ‘system development life cycle’, which include: assessment of user needs and setting/context of use; development of system components; testing system and tasks with users; testing system and tasks with users within context/setting; and routine use [34]. Reviewed studies evaluated these to different extents. Many reported conducting preliminary research assessing end-users’ needs to identify information and functional requirements of HIT programs [15, 23, 24, 27, 28, 30]. Most studies reported programs were then designed incorporating the perspectives of these end-users [15, 21, 22, 28]. All studies tested the system and tasks with users within the hospital setting. As no studies had yet implemented programs for routine use, this was not reported.

Whilst most studies reported a user-centred approach to designing HIT programs, they didn’t always associate this with outcomes. Also, they did not differentiate between aspects of user-centred design or explicitly refer to stages of evaluation throughout the system development life cycle. This may suggest the theoretical underpinnings of HIT usability are not well understood by health researchers, impacting the design of usability studies and highlighting the importance of meaningful partnerships between health and IT experts in conception, design and evaluation of HIT usability studies.

**Proposition 5** *Supporting patients in the use of HIT, including familiarisation, training and ongoing support is critical to patients’ acceptance, engagement and use of this technology. Patients acknowledge that both HCPs and HIT have unique but complementary roles, and both are important for enabling participation in care.*

Nearly all studies described some type of support provided to patients in the use of HIT programs, however details about how patients were supported were lacking in most studies. Most reported providing a short orientation to the program to familiarise patients with its use [15, 21, 22, 27, 31]. Some studies described this as a ‘brief’ or ‘very brief’ ‘explanation’, ‘orientation’ or ‘training session’, without indicating the actual time spent with participants [21–23, 27], while others indicated tutorials or instructions lasted for five minutes or less [15, 31]. Some simply reported that patients were ‘encouraged to use the application’ [16] or that ‘nurses instructed patients’ in its use [32]. Others provided more in-depth training, lasting around 15 min per patient [24, 30]. Two studies did not report patient training [26, 29].

Studies providing patients with support in program use reported outcomes such as high patient satisfaction [21–24, 27, 30]; perceived ease of use [21–24, 31]; and engagement in or use of programs by patients [15, 21–23, 26, 32]. However, these outcomes were not linked with support provided (i.e. no proposed mechanisms). As noted in the previous section, these outcomes are likely due to a combination of mechanisms or propositions, so it is difficult to determine the extent to which each one contributed. In several studies, patients mentioned the program was easy to use, which may be linked with the support provided.

Patients often highlighted the importance of receiving support in HIT use and having interactions with HCPs. Patients were generally satisfied with HIT programs, found them useful, and appreciated the time and information provided, as well as the ability to learn at their own pace. However, they still wished to maintain relationships with HCPs and did not want programs to replace HCPs. Rather, they thought the technology could support staff in providing quality patient care. It seems that whilst patients enjoy using HIT to participate in their care, they still want to engage with HCPs in using this technology.

## Discussion

This review identified a number of key features of interventions using technology to engage patients in their hospital care. These included information sharing, self-assessment and feedback, tailored education, user-centred design, and support in the use of HIT; which all fell under an overarching theme ‘interactive learning’. For each of these features, a proposition was developed to explain how and why they facilitated patient participation in care with consideration of context, mechanisms and outcomes. This review provides important insight into what intervention strategies work, how and why, for whom and under what conditions, from both health and IT perspectives; to inform future design of interventions using technology to engage patients in their hospital care.

Overall there was a stronger representation of health than IT disciplines in the studies reviewed. That is, most studies were written from a health perspective and underpinned by theories or concepts grounded in health (i.e. patient-centred care, patient participation, adult learning theories and behaviour change theories). No studies reported on theories of HIT usability or interface design principles; which are vital to consider for enabling patient engagement with these technologies. Propositions with a health focus (propositions 1–3) were more developed, as papers described these features in more detail; whereas for IT-related propositions (4 and 5) there was less information available in papers to determine the CMO relationship. This highlights the need for interdisciplinary collaboration in the design and conduct of HIT usability studies.

The strong underpinning of patient participation theory in studies reviewed was manifest in the propositions developed, which reflected all four aspects of participation identified in a concept analysis by Sahlsten et al. [35]. Firstly, ‘meaningful exchange of information and knowledge between patients and HCPs’ was apparent in all studies through information sharing, assessment and feedback, and tailored education. Patient participation was enabled by HIT interventions allowing patients to access and/or enter their own health information and providing individually adapted information/knowledge to patients [35]. Second, the importance of ‘an established relationship between the patient and HCP’ was particularly emphasised in the proposition around support in the use of HIT. Patients expressed whilst they were highly satisfied with HIT interventions, they wished to maintain a relationship and contact with their HCPs in using this technology. Some studies also indicated improved communication between patients and HCPs with HIT interventions. Third, ‘shared decision making’ was an underlying mechanism in several propositions and was a facilitator to patient participation in care (Fig. 2). Several aspects of HIT interventions promoted or enabled shared decision making as a way to participate in care. Finally, ‘surrendering of some power or control by HCPs’ was evident in the overall use of HIT and interactive learning, where patients took responsibility for the content, extent and pace of learning. Programs using information sharing also allowed patients to be the gatekeepers of their health information, giving some control back to the patient. This aspect of participation is vital for empowering patients to take responsibility and participate in self-care and self-management.

Adult learning and behaviour change theories were also evident in the findings of this review. Interactive learning, and in particular, tailored education, resonate with the core principles of Knowles’ adult learning theory, andragogy, which postulates that individuals’ orientation and readiness to learn are life-related; that is, they are ready to learn when they experience an event (such as an illness) or need to learn to cope with a real life situation [36]. This review found patients appreciated information most when it was relevant, specific to their needs and delivered in a timely manner (i.e. ‘just in time’ education during admission for a particular health condition [26]). Andragogy also suggests individuals’ prior experiences influence their learning; that adults define themselves by their experiences, and will respond positively to education that acknowledges and values these experiences [36]. This was particularly evident in studies where patients highlighted the importance of contextual and empathic tailored educational content that resonated with their own life, situation, and experiences with their disease [21–23]. The proposed mechanisms underpinning increased patient participation also aligns with Bandura’s theory of self-efficacy; strategies such as tailored education and self-assessment and feedback empowered patients and built confidence in their ability to participate in health tasks and achieve health goals [37].

Consideration of HIT usability and interface design was rudimentary in studies reviewed, suggesting an insufficient representation of the IT discipline in intervention design and evaluation. The major limitations of studies included a lack of theoretical underpinning from an IT perspective, inadequate reporting on evaluation of each system development life cycle phase, and a singular and broad usability evaluation focus; congruent with findings of a previous review of 629 IT usability studies [34]. Usability is defined as the extent to which a program can be used by a specified population to achieve goals with effectiveness, efficiency and satisfaction within a certain context [34]. It is an important predictor of acceptance and ability to use technology-based interventions effectively and as intended, making usability a necessary component of HIT design [38, 39]. Lack of attention to HIT design and evaluation may affect usability and result in reduced efficiency, effectiveness and satisfaction of interventions [34].

Reviewed studies only tended to consider usability in a holistic sense (i.e. overall patient satisfaction or use) and did not explore specific components of usability. Broadly, there are two separate aspects to human computer interfaces that impact usability; how information is presented to users (presentation design) and how users interact with the system (interaction design) [40]. Presentation design refers to the use of style, colour coding, metaphors and conceptual models, whilst interaction design is about interaction modalities and styles, for example question-answer, menu selection, form filling etc. [40]. Reviewed studies did not differentiate between these aspects of user interface design. Furthermore, the seven general user interface design principles that mediate usability: learnability, user familiarity, consistency, minimal surprise, recoverability, user guidance and user diversity [41] were not reported in studies. The theoretical underpinnings of HIT usability were not apparent in studies and this impacted on their design, including the methods used and outcomes assessed. The complexity of health care environments and HIT interventions, and the demand for tailored systems to meet patients’ needs make usability engineering methods critically important [42]. Researchers must form interdisciplinary partnerships when designing and evaluating HIT interventions and their usability.

Patients’ ability to access technology in the hospital setting is an important consideration when designing HIT. Some studies failed to consider patient populations who might not be able to use HIT, which impacts on the utility of their programs and raises questions about equity and equality of care [43]. Others acknowledged their program could only be used by English speaking patients who were cognitively intact [24, 27, 30, 32] and well enough to participate [16]; most authors acceded this was an area for future work. Several studies considered patients with motor, vision or hearing impairments, and adapted HITs accordingly [21, 26]. A few authors acknowledged that technology may actually improve access to health information for some patients through thoughtful interface design (larger text, easy to press buttons, and headphones or volume control) and convenient mediums such as handheld devices [15, 16, 26, 28, 30]. Some found even patients with limited experience with technology could use programs easily [24].

There are some limitations to this review. The outcomes of the review are based on relatively small studies from an emerging body of literature. There is the potential that some studies were missed and not all information was represented; however we included all studies that we found that met inclusion criteria, and data saturation was reached. Due to the small number of studies using technology to engage hospitalised patients in their care, we could not focus on one type of condition or aspect of care; however including studies from a variety of contexts may increase the usefulness of the review. It is possible that the experience and background of the reviewer could affect interpretation of findings; however we tried to enhance trustworthiness by having frequent discussions among the study team, which was multi- and inter-disciplinary (importantly containing health and IT researchers).

## Conclusions

HIT interventions have great potential for engaging hospitalised patients in their care. This review found patient participation is facilitated by interactive learning, which is underpinned by strategies from both health and IT perspectives (information sharing, self-assessment and feedback, tailored education, user-centred design, support in use of HIT). As all studies included in this review used a mix of these strategies, it is difficult to pinpoint which are most important for engaging patients in their care. However, it is likely that a combination of these would be most effective, as each contributes to patient acceptability and use in different ways. Overall, studies had a strong health focus but lacked depth from an IT perspective in reporting HIT intervention development and testing. Interdisciplinary collaboration between health and IT researchers is vital for effective design and evaluation of HIT interventions.

## Abbreviations

CMO: Context, mechanism, outcome
HCP: Health care professional
HIT: Health information technology
IT: Information technology
RCT: Randomised control trial
