# Erratum to: Using technology to engage hospitalised patients in their care: a realist review

**DOI:** 10.1186/s12913-017-2577-5

**Published:** 2017-09-13

**Authors:** Shelley Roberts, Wendy Chaboyer, Ruben Gonzalez, Andrea Marshall

**Affiliations:** 1 NHMRC Centre of Research Excellence in Nursing, Menzies Health Institute Queensland, Griffith University, Gold Coast Campus, Gold Coast, QLD 4222 Australia; 2 School of Information and Communication Technology, Griffith University, Gold Coast Campus, Gold Coast, QLD 4222 Australia; 3 School of Nursing and Midwifery, Menzies Health Institute Queensland, Griffith University, Gold Coast Campus, Gold Coast, QLD 4222 Australia; 4 Nursing and Midwifery Education and Research Unit, Gold Coast University Hospital, Southport, QLD 4215 Australia

## Erratum

Following publication of the original article [1], one of the authors reported that she had requested in the proofs that a Figure to be moved from the Results section to the Methods section, where it belonged. However - this was not done and the paper was published with the figure in the wrong section.

The original article has been corrected.
